# Impact of coronary plaque geometry on plaque vulnerability and its association with the risk of future cardiovascular events in patients with chest pain undergoing coronary computed tomographic angiography—the GEOMETRY study: Protocol for a prospective clinical trial

**DOI:** 10.1097/MD.0000000000013498

**Published:** 2018-12-10

**Authors:** Mihaela Ratiu, Monica Chitu, Imre Benedek, Theodora Benedek, Istvan Kovacs, Nora Rat, Ciprian Rezus

**Affiliations:** a Department of Radiology; b Department of Cardiology, University of Medicine, Pharmacy, Sciences and Technology of Targu Mures; c Department of Advanced Research in Multimodality Cardiovascular Imaging, Cardio Med Medical Center, Targu Mures; d Department of Medical Clinic III - Internal Medicine, University of Medicine and Pharmacy ‘Gr.T.Popa’, Iasi, Romania.

**Keywords:** atheromatous plaque, coronary computed tomography angiography, coronary lesion geometry, vulnerable plaque

## Abstract

**Introduction:**

Coronary computed tomography angiography (CCTA) has emerged as a valuable noninvasive imaging tool for assessing atheromatous plaque morphology and composition, and several CCTA features have been validated as reliable indicators of the plaque-associated risk. However, the role of lesion geometry as a CCTA feature of plaque vulnerability has not been investigated so far.

**Material and methods:**

Here we present the study protocol of the GEOMETRY trial, a prospective, single center, cohort study in which we aim to investigate the relationship between plaque geometry (as expressed by cross-sectional and longitudinal plaque eccentricity) and the risk for major adverse cardiac events (MACE) during 2 years of follow-up, in order to validate plaque eccentricity as a new CCTA marker of coronary plaque vulnerability. One thousand patients with suspected coronary artery disease (CAD) and pretest probability of CAD between 15% and 85%, who undergo CCTA and in whom CCTA identifies the presence of at least 1 significant coronary plaque (producing a luminal narrowing of at least 50%) will be enrolled in the study. Based on the results of complex image post-processing and plaque analysis, patients will be divided into 2 groups: group 1—patients in whom CCTA analysis identifies only non-eccentric coronary plaque; and group 2—patients in whom CCTA analysis reveals the presence of at least 1 eccentric significant coronary plaque producing a significant luminal narrowing. Study outcomes will consist in the rate of major cardiovascular events and the rate of plaque progression during follow-up.

The study is funded by the Romanian Ministry of European Funds, the Romanian Government and the European Union, as part of the research grant number 103544/2016 – PlaqueIMAGE (contract number 26/01.09.2016).

**Conclusion:**

In conclusion, GEOMETRY will be the first CCTA-based study that will investigate the impact of geometric distribution of coronary atheromatous plaque on the future risk of cardiovascular events and on the rate of plaque progression, introducing and validating a new potential feature of plaque vulnerability represented by plaque geometry.

## 1 Introduction

### 1.1 Background and rationale

Ischemic heart disease represents the leading cause of mortality worldwide, causing 9.4 million of deaths in 2016.^[1]^ Atherosclerosis represents the underlying condition leading to myocardial ischemia, ultimately resulting in acute coronary syndromes (ACS) and having devastating consequences on public health. The vast majority of ACSs are the consequence of a sudden modification in coronary plaque morphology leading to plaque rupture or erosion, the 2 principal mechanisms triggering an acute coronary event.^[2]^ Atheromatous plaques that are prone to rupture or erosion following a complex process of transformation and alteration of plaque composition are known as vulnerable coronary plaques and can be easily identified nowadays with the help of modern imaging techniques.^[3,4]^ Due to the recent progress in the field of coronary computed tomography angiography (CCTA), it has emerged as a valuable noninvasive imaging tool for assessing atheromatous plaque morphology and composition. Moreover, CCTA-based imaging-derived markers associated with vulnerability have been validated as reliable indicators of the plaque-associated risk. Such key CCTA features of plaque vulnerability identified by previous studies include positive remodeling (PR), low-attenuation plaque (LAP), napkin-ring sign (NRS), and spotty calcifications (SC).^[5,6]^ Interestingly, intravascular ultrasound studies demonstrated that ruptured coronary plaques are usually eccentric.^[7]^ A combined intravascular ultrasound (IVUS) and optical coherence tomography (OCT)-based study reported that lesions demonstrating plaque erosion had a greater plaque eccentricity index than those with plaque rupture or calcified nodules (*P* < .001 and *P* < .001).^[8]^ At the same time, progression of atherosclerotic lesions was more frequent in eccentric lesions than in concentric ones.^[7,9]^ However, the role of lesion eccentricity as a CCTA feature of plaque vulnerability has not been investigated so far. Furthermore, the impact of lesion geometry, as defined by the type of eccentricity (transversal or longitudinal), on the degree of vulnerability is unclear.

Here we present the study protocol of the GEOMETRY trial, a prospective, single center, cohort study in which we aim to investigate the relationship between plaque geometry (as expressed by cross-sectional and longitudinal plaque eccentricity), on one hand, and the risk for major adverse cardiac events (MACE) during 2 years of follow-up, in order to validate plaque eccentricity as a new CCTA marker of coronary plaque vulnerability.

### 1.2 Study objectives

The primary objective of the study is to evaluate the association between different patterns of plaque geometry and the risk for major adverse cardiac events MACE (defined as all-cause mortality, cardiovascular death, myocardial infarction, repeated revascularization, repeated hospitalizations for cardiovascular related incidents, cerebrovascular events) during a 2-year follow-up.

The secondary objective of the study is to evaluate the association of plaque eccentricity with plaque vulnerability and plaque progression after 2 years of follow-up.

## 2 Methods/design

### 2.1 Study design

GEOMETRY is a prospective, non-randomized, cohort, single-center study to investigate the relationship between plaque eccentricity, plaque vulnerability, and the risk for MACE in order to validate plaque eccentricity as a new CCTA marker of coronary plaque vulnerability.

### 2.2 Ethics

The study protocol was approved by the Ethics Committee for Scientific Research of the University of Medicine and Pharmacy of Tirgu Mures (certificate of approval: 352/13.12.2017) and the Ethics Committee for Scientific Research of the Cardio Med Medical Center (certificate of approval 27/12.02.2017). All study procedures comply with the Declaration of Helsinki of 1975, and all patients will sign an informed consent prior to enrollment in the study.

### 2.3 Study population

GEOMETRY will be a non-randomized, observational, single-center study including patients with chest pain and pre-test probability of coronary artery disease between 15% and 85%, referred for CCTA (according to the recommendation of the guidelines of the European Society of Cardiology).^[10]^

Inclusion criteria:

- Patients with suspected coronary artery disease (CAD) and pre-test probability of CAD between 15% and 85%, who undergo CCTA, and in whom CCTA will identify presence of at least 1 significant coronary plaque (producing a luminal narrowing of at least 50%).
- Ability to provide informed consent.
- Age over 18 years.

Exclusion criteria:

- Patients with pre-test probability of CAD >85% or <15%.
- Electrocardiographic evidence of ST-segment elevation acute myocardial infarction.
- Presence of pre-existing CAD including prior myocardial infarction.
- History of coronary artery revascularization (by percutaneous coronary intervention, stent, or bypass graft surgery).
- Atrial fibrillation or other irregular rhythm.
- Unwillingness or incapacity to provide informed consent.
- Allergy to iodine contrast media.
- Inability to tolerate beta-blocker medication.
- Renal insufficiency (serum creatinine values higher than 1.5 mg/dL) or renal failure requiring dialysis.
- Pregnant women or lactation.
- Active malignancy or malignancy within the last 5 years prior to enrolment.
- Conditions associated with an estimated life expectancy of under 2 years.
- Coronary calcium score >1000.

### 2.4 Study settings

The study will be conducted in the Center of Advanced Research in Multimodality Cardiac Imaging of the Cardio Med Medical Center, being funded by the Romanian Ministry of European Funds, the Romanian Government and the European Union, as part of the research grant number 103544/2016—PlaqueIMAGE (contract number 26/01.09.2016), which was selected for funding following an international peer-review procedure within the national competition of research grants in.

### 2.5 Study groups

One thousand patients who meet the selection criteria will be included in the trial and will be divided into 2 groups, namely patients in whom screening CCTA analysis identifies only non-eccentric coronary plaque (group 1) and patients in whom CCTA analysis reveals the presence of at least 1 eccentric significant coronary plaque producing a significant luminal narrowing (group 2).

### 2.6 Study procedures and outcome assessment

In all patients clinical data including sex, age, comorbidities, history of coronary artery disease, stroke, peripheral arterial disease, diabetes, smoking status, as well as clinical status and laboratory tests (creatinine, total cholesterol, low-density lipoprotein [LDL]-cholesterol, triglycerides) will be recorded. All patients will undergo CCTA scanning of the coronary arteries at screening and will be enrolled in the study if CCTA reveals the presence of at least 1 obstructive coronary plaque in any coronary artery. In randomized patients, CCTA scanning will be followed by complex image postprocessing for assessment of plaque composition, morphology, and geometry, using available research software for plaque reconstruction and analysis.

#### 2.6.1 CCTA scanning protocol

CCTA will be performed with a 128-slices single source CT scanner with retrospective electrocardiographic gating, at a tube voltage of 100 kV, a gantry rotation time of 330 ms and a collimation of 128 × 0.6. Oral beta-blockers will be administered to all patients with a heart rate >65 beats/min in order to achieve the desired heart rate, and 0.4 mg nitroglycerin will be administered sublingually 2 minutes before scanning in order to obtain a coronary vasodilatation and thus a superior image quality. Contrast agent will be injected with a flow rate of 5 mL/s in the antecubital vein, using an18-gauge venous catheter placed in the right cubital fossa, with a total contrast quantity between 80 and 100 mL adapted to patient body weight. Contrast administration will be followed by a flush of 50 mL saline solution with the same flow rate. CT datasets will be retrospectively reconstructed with a slice thickness of 0.6 using a medium soft-tissue convolution kernel and the phase with the fewest motion artifacts and best image quality will be selected for image analysis and for atheromatous plaques assessment.

#### 2.6.2 Plaque reconstruction and analysis

All reconstructed CCTA images will be further evaluated semi automatically using a three-dimensional (3D) contour detection algorithm (Syngo.via Multimodality Workplace, Siemens, Frontier–Coronary Plaque Analysis platform, Siemens, Erlangen, Germany). All coronary vessels with a diameter of at least 2 mm will be assessed using the 19 coronary segments model.

Quantitative evaluation of the atheromatous lesions will include plaque length, vessel volume, lumen volume, plaque volume, and the severity of luminal narrowing. The volumetric measurements will be performed using as reference the proximal and the distal extremities of the plaques, and assessment of plaque composition will include determination of dense calcium and non-calcified plaque components, such as lipid rich and fibrotic tissue.

Qualitative assessment of plaque characteristics will investigate the presence of vulnerability features inside the coronary plaque: PR, LAP, SC, and NRS.

#### 2.6.3 Study definitions

In this study, a significant coronary plaque is defined as a plaque producing at least 50% luminal narrowing. The remodeling index (RI) represents the ratio between the cross-sectional area at the site of maximum stenosis divided by the average of the proximal and distal reference cross-sectional areas, and PR is defined as a remodeling index of 1.1 or greater.^[11]^ Plaques having at least 10% of their content non-calcified, with a CT density of <30 HU will be classified as LAP.^[12]^ SC are defined by a small dense area (>130 HU) surrounded by non-calcified plaque tissue.^[13]^ NRS will be considered present following identification of a non-calcified plaque with a central area of low attenuation in contact with the lumen, and a ring-like higher attenuation tissue, surrounding the central area.^[11,14]^

Cross-sectional plaque eccentricity will be assessed according to the location of the circulant lumen in a transverse section at the level of maximum stenosis. The cross-sectional eccentricity (CE) index will be calculated as follows: (maximum wall thickness – minimum wall thickness)/maximum wall thickness. Eccentric lesions will be defined as having a CE index of 0.3 or greater, whereas concentric lesions will be defined as having a CE index of <0.3. Longitudinal plaque eccentricity, defined as stenosis with an abrupt narrowing of the proximal or distal edge, will be assessed according to the location of maximum stenosis in a longitudinal section. The longitudinal eccentricity (LE) index will be calculated as the ratio of distance between the proximal end of the lesion and the place of maximum stenosis to the lesion length.^[15]^ Lesions with an LE index of ≤0.4 will be classified as descendent; lesions with an LE index of ≥0.6 will be classified as ascendant; and intermediate lesions with an LE index between 0.4 and 0.6 will be classified as non-eccentric plaques.

### 2.7 Study time

The clinical trial will be conducted from November 2018 to August 2019, followed by a 24-months follow-up.

### 2.8 Outcomes

The primary outcome of the study is represented by the rate of MACE during follow-up. Secondary outcome refers to coronary plaque vulnerability associated to plaque eccentricity, based on the number of vulnerability markers in eccentric versus concentric plaques.

### 2.9 Participation timeline

Screening (Day 0)

- Obtain and document consent from participant on study consent form.
- Verify inclusion/exclusion criteria.
- Imaging: 128-multislice CT angiography scanning and basic plaque assessment—presence of coronary plaque and degree of luminal narrowing.

Baseline (day 0):

- Obtain demographic information, medical history, medication history, alcohol, and tobacco use history.
- Record results of physical examinations and 12-lead ECG.
- Collect blood specimens.
- Imaging: image post-processing for complex plaque assessment—plaque morphology, composition, and geometry

Visit 1 (month 6):

- Record results of physical examinations, 12-lead ECG, and medical history.
- MACE assessment.

Visit 2 (month 12):

- Record results of physical examinations, 12-lead ECG, and medical history.
- MACE assessment.

Visit 3 (month 18):

- Record results of physical examinations, 12-lead ECG, and medical history.
- MACE assessment.

Final study visit (month 24):

- Record results of physical examinations, 12-lead ECG, and medical history.
- Repeat CCTA and plaque assessment.
- End-point evaluation.

**Figure 1:**
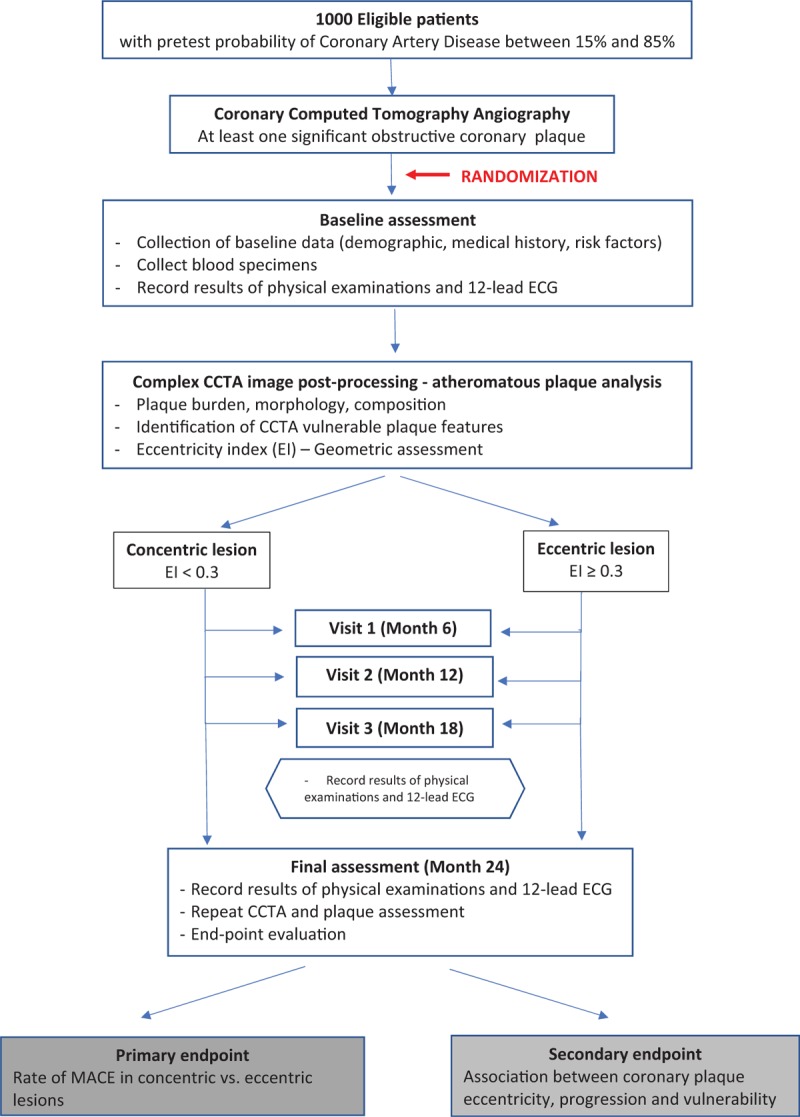
Flowchart diagram of GEOMETRY study.

Figure 1 illustrates the GEOMETRY diagram with the flowchart that will be used in the study.

### 2.10 Sample size

The study will include 1000 subjects with suspected CAD who are referred to CCTA by the attending physician and in which presence of at least 1 coronary plaque is confirmed by CCTA. After analysis of coronary plaques eccentricity index, patients will be distributed in 2 groups according to the presence or absence of significant eccentric lesions in coronary arteries.

Sample size calculation was performed using the StatMate 2.0 software (GraphPad Software, San Diego, CA). For sample calculation, the proportion of event-free population was estimated at 90%. According to this calculation, a sample size of 500 patients in each subgroup has a 90% power to detect an increase in MACE-free rates proportion of 0.054, with a significance level (alpha) of 0.05 (two-tailed). Therefore, the total sample size was established at 1000 patients.

### 2.11 Statistical analysis

Statistical analysis will be performed using Graph Pad InStat 3.10 software (GraphPad Software, San Diego, CA) at the level of significance 5%. All data will be checked for normality. Continuous variables with normal distribution will be presented as mean ± standard deviation and will be compared using *t* test. Non-normally distributed variables will be analyzed using the Mann–Whitney *U* test. Categorical variables will be expressed in numbers and percentages and will be compared using the Fisher exact test.

## 3 Discussion

The study describes the protocol for a prospective cohort, single-center study which aims to study the correlation between coronary plaque eccentricity, lesion vulnerability, and 24-months MACE rates in patients with suspected coronary artery disease referred for CCTA for coronary luminal evaluation.

The main contribution of this study is to incorporate the evaluation of coronary plaque eccentricity in routine analysis during coronary computed tomography angiography in order to identify lesions with a more vulnerable phenotype using a noninvasive method.

In this study we hypothesize that lesion geometry has an impact on plaque vulnerability and could be associated with several well-established vulnerability features such as SC, LAP, presence of non-calcified plaques, and lipid-rich atheroma.

Previous postmortem studies demonstrated that plaque rupture and erosion occur more frequently in eccentric atheroma and launched the hypothesis of plaque eccentricity as a feature significantly associated with plaque vulnerability.^[16]^ Costopoulos et al^[17]^ recently demonstrated that plaque rupture occurs in the regions exposed to increased plaque structural stress, which is determined by plaque composition, architecture, and geometry. In their study, the authors reported that plaque structural stress increases with lumen eccentricity (*r* = 0.32, *P* = .001). It has been suggested that hemodynamic forces acting on vascular endothelium can initiate the atherosclerotic process and a pro-atherogenic shear stress profile can be associated with distinct transformation of plaque phenotype toward increased vulnerability.^[18–20]^ At the same time, endothelial shear stress is directly associated with plaque geometry, a baseline low endothelial shear stress being an independent predictor of substantially increasing plaque eccentricity in the study of Papafaklis et al^[21]^ (odds ratio [OR] = 2.33, *P* = .003) and in the study of Puri et al.^[22]^ This shows a complex inter-relation between plaque geometry, hemodynamic profile, and plaque vulnerability.

However, to the best of our knowledge, GEOMETRY will be the first CT-based study investigating a potential direct association between plaque geometry and vulnerability features. Our hypothesis is that plaque eccentricity, exposing the atheroma to an increased circumferential stress, may be responsible for plaque rupture.

Various recent studies reported the role of CT-derived features for characterization of the functional significance of a coronary plaque.^[23,24]^ A recent study published by Kang et al^[15]^ demonstrated that coronary lesion geometry has a direct impact on the functional significance of a coronary stenosis, showing that complex coronary lesions are independently associated with reduced fractional flow reserve (FFR) values and that lesion eccentricity is the most significant independent predictor for low FFR. In the GEOMETRY study presented here, we extended this hypothesis to investigate for the first time the impact of lesion geometry on plaque vulnerability and the association between geometric distribution of atheromatous plaques and the related risk of future cardiovascular events.

## 4 Conclusions

In conclusion, GEOMETRY will be the first CCTA-based study that will investigate the impact of geometric distribution of coronary atheromatous plaque on the future risk of cardiovascular events and on the rate of plaque progression, introducing and validating a new potential feature of plaque vulnerability represented by plaque geometry.

## Author contributions

All authors have been involved in all stages of the study design and have participated in writing the protocol.

Submission to ethical committee was done by Mihaela Ratiu.

Mihaela Ratiu is the radiologist performing the CCTA imaging interpretation.

Mihaela Ratiu, Nora Rat and Theodora Benedek will be involved in trial statistical analysis and interpretation.

**Conceptualization:** Mihaela Ratiu, Monica Chitu, Imre Benedek, Theodora Benedek, Istvan Kovacs, Nora Rat, Ciprian Rezus.

**Data curation:** Theodora Benedek, Istvan Kovacs.

**Formal analysis:** Mihaela Ratiu, Theodora Benedek, Nora Rat.

**Investigation:** Mihaela Ratiu, Theodora Benedek.

**Methodology:** Mihaela Ratiu, Imre Benedek, Theodora Benedek, Ciprian Rezus.

**Resources:** Imre Benedek.

**Supervision:** Imre Benedek, Theodora Benedek.

**Visualization:** Ciprian Rezus.

**Writing – original draft:** Mihaela Ratiu, Monica Chitu, Imre Benedek, Theodora Benedek, Istvan Kovacs, Nora Rat, Ciprian Rezus.

## References

[1] Global Health Estimates 2016: Deaths by Cause, Age, Sex, by Country and by Region, 2000-2016. Geneva: World Health Organization; 2018.

[2] Crea F Libby P Acute coronary syndromes. The way forward from mechanism to precision treatment. Circulation 2017;136:1155–66. 28923905 10.1161/CIRCULATIONAHA.117.029870 PMC5679086

[3] Stefandis C Antoniou CK Tsiachris D Coronary atherosclerotic vulnerable plaque: current perspectives. J Am Heart Assoc 2017;6:pii: e005543. 10.1161/JAHA.117.005543 PMC5524044 28314799

[4] Nyulas T Marton E Rus VA Morphological features and plaque composition in culprit atheromatous plaques of patients with acute coronary syndromes. J Cardiovasc Emerg 2018;4:84–94.

[5] Pozo E Agudo-Quilez P Rojas-Gonzales A Noninvasive diagnosis of vulnerable coronary plaque. World J Cardiol 2016;8:520–33. 27721935 10.4330/wjc.v8.i9.520 PMC5039354

[6] Benedek T Gyongyosi M Benedek I Multislice computed tomographic coronary angiography for quantitative assessment of culprit lesions in acute coronary syndromes. Can J Cardiol 2013;29:364–71. 23333164 10.1016/j.cjca.2012.11.004

[7] Yamagishi M Terashima M Awano K Morphology of vulnerable coronary plaque: insights from follow-up of patients examined by intravscular ultrasound before an acute coronary syndrome. J Am Coll Cardiol 2000;35:106–11. 10636267 10.1016/s0735-1097(99)00533-1

[8] Higuma T Soeda T Abe N A combined optical coherence tomography and intravascular ultrasound study on plaque rupture, plaque erosion, and calcified nodule in patinets with st-segment elevation myocardial infarction: incidence, morphologic characteristics, and outcomes after percutaneous coronary intervention. JACC Cardiovasc Interv 2015;8:1166–76. 26117464 10.1016/j.jcin.2015.02.026

[9] Ohara T Toyoda K Otsubo R Eccentric stenosis of the carotid artery associated with ipsilateral cerebrovascular events. AJNR Am J Neuroradiol 2008;29:1200–3. 18339721 10.3174/ajnr.A0997 PMC8118845

[10] Montalescot G Sechtem U Achenbach S ESC guidelines on the management of stable coronary artery disease: The Task Force on the management of stable coronary artery disease of the European Society of Cardiology. Eur Heart J 2013;34:2949–3003. 23996286 10.1093/eurheartj/eht296

[11] Maurovich-Horvath P Ferencik M Voros S Comprehensive plaque assessment by coronary CT angiography. Nat Rev Cardiol 2014;11:390–402. 24755916 10.1038/nrcardio.2014.60

[12] Benedek T Jako B Benedek I Plaque quantification by coronary CT and intravascular ultrasound identifies a low CT density core as a marker of plaque instability in acute coronary syndromes. Int Heart J 2014;55:22–8. 24463925 10.1536/ihj.13-213

[13] Motoyama S Kondo T Sarai M Multislice computed tomographic characteristics of coronary lesions in acute coronary syndromes. J Am Coll Cardiol 2007;50:319–26. 17659199 10.1016/j.jacc.2007.03.044

[14] Otsuka K Fukuda S Tanaka A Napkin-ring sign on coronary CT angiography for the prediction of acute coronary syndrome. JACC Cardiovasc Imaging 2013;6:448–57. 23498679 10.1016/j.jcmg.2012.09.016

[15] Kang DY Ahn JM Kim YK Impact of coronary lesion geometry on fractional flow reserve: data From International Cardiology Research In-Cooperation Society-Fractional Flow Reserve and Intravascular Ultrasound Registry. Circ Cardiovasc Imaging 2018;11:e007087. 29895713 10.1161/CIRCIMAGING.117.007087

[16] Jain S Biligi D An autopsy study on coronary atherosclerosis with morphological and morphometric analysis. Int J Sci Res 2013;4:1522–6.

[17] Costopoulos C Huang Y Brown AJ Plaque rupture in coronary atherosclerosis is associated with increased plaque structural stress. JACC Cardiovasc Imaging 2017;10:1472–83. 28734911 10.1016/j.jcmg.2017.04.017 PMC5725311

[18] Li F McDermott MM Li D The association of lesion eccentricity with plaque morphology and components in the superficial femoral artery: a high-spatial-resolution, multi-contrast weighted CMR study. J Cardiovasc Magn Reson 2010;12:37. 20591197 10.1186/1532-429X-12-37 PMC2904754

[19] Rothwell PM Gibson R Warlow CP Interrelation between plaque surface morphology and degree of stenosis on carotid angiograms and the risk of ischemic strike in patients with symptomatic carotid stenosis. Stroke 2000;31:615–21. 10700494 10.1161/01.str.31.3.615

[20] Kaazempur-Mofrad MR Wada S Myers JG Mass transport and fluid flow in stenotic arteries: axisymmetric and asymmetric models. Int J Heat Mass Transf 2005;48:4510–7.

[21] Papafaklis MI Takahashi S Antoniadis AP Effect of the local hemodynamic environment on the de novo development and progression of eccentric coronary atherosclerosis in humans: insights from PREDICTION. Atherosclerosis 2015;240:205–11. 25801012 10.1016/j.atherosclerosis.2015.03.017

[22] Puri R Leong DP Nicholls SJ Coronary artery wall shear stress is associated with endothelial dysfunction and expansive arterial remodeling in patients with coronary artery disease. EuroIntervention 2015;10:1440–8. 24425248 10.4244/EIJV10I12A249

[23] Zhang JM Shuang D Baskaran L Advanced analyses of computed tomography coronary angiography can help discriminate ischemic lesions. Int J Cardiol 2018;267:208–14. 29685695 10.1016/j.ijcard.2018.04.020

[24] Orzan M Dobra M Chitu M A comparative preliminary study on CT contrast attenuation gradient versus invasive FFR in patients with unstable angina. J Cardiovasc Emerg 2017;3:71–8.

